# Simultaneous measurement of duloxetine hydrochloride and avanafil at dual-wavelength using novel ecologically friendly TLC-densitometric method: application to synthetic mixture and spiked human plasma with evaluation of greenness and blueness

**DOI:** 10.1186/s13065-024-01195-2

**Published:** 2024-05-03

**Authors:** Sayed M. Derayea, Hadeer A. Elhamdy, Mohamed Oraby, Khalid M. Badr El-Din

**Affiliations:** 1 Analytical Chemistry Department, Faculty of Pharmacy, Minia University, Minia, 61519 Egypt; 2 Department of Pharmaceutical Analytical Chemistry, Faculty of Pharmacy, Sohag University, Sohag, 82524 Egypt

**Keywords:** Duloxetine, Avanafil, HPTLC-Densitometry, Dual-wavelength, Human plasma, Greenness and blueness

## Abstract

The simultaneous assay of duloxetine hydrochloride (DLX) and avanafil (AVN) in their pure forms, synthetic mixtures, and spiked human plasma was achieved using a novel, eco-friendly, sensitive, and specific HPTLC methodology that have been established and validated. Measuring the levels of co-administered antidepressants and sexual stimulants in biological fluids is an important step for individuals with depression and sexual problems. Separation was performed successfully using pre-coated silica gel 60-F254 as a stationary phase and a mobile phase composed of methanol, acetone, and 33% ammonia (8:2:0.05, v/v/v). Compact bands were produced by the optimized mobile phase that was chosen for development (Rf values were 0.23 and 0.75 for DLX and AVN, individually) after dual-wavelength detection for DLX and AVN at 232 and 253 nm, respectively. The results of polynomial regression analysis were exceptional (*r* = 0.9999 for both medicines) over concentration ranges of 5-800 and 10-800ng/spot for DLX and AVN, respectively. The quantitation limits were 4.69 and 9.53 ng/spot (0.31 and 0.94 µg/mL), whereas the detection limits were 1.55 and 3.15 ng/spot (0.63 and 1.91 µg/mL), for DLX and AVN, respectively. The International Council for Harmonization (ICH) criteria served as the basis for validating the established approach. Moreover, the proposed technique was evaluated in terms of greenness using four contemporary ecological metrics: The Analytical Greenness software (AGREE), the Green Analytical Procedure Index (GAPI), Eco-Scale, and the National Environmental Method Index (NEMI). Additionally, the Blue Applicability Grade Index (BAGI), a newly developed tool for evaluating the practicality (blueness) of procedures, was taken into consideration when evaluating the sustainability levels of the established approach.

**Supplementary Information:**

The online version contains supplementary material available at 10.1186/s13065-024-01195-2.

## Introduction

**Fig. 1:**
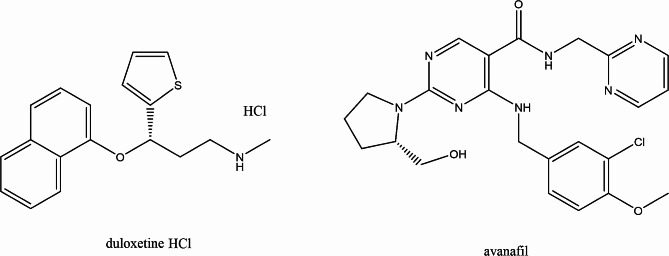
The chemical structures of the studied drugs

Duloxetine hydrochloride is an antidepressant drug with the chemical name (+) - (S) - N-methyl -(gamma)- (1- naphthyloxy) -2-thiophenepropylamine hydrochloride1 (Fig. 1) [1]. Duloxetine hydrochloride received FDA approval in 2004 for the therapeutic management of major depressive disorder (MDD), pain triggered as a result of Diabetes-related neuropathy in the peripheral nerves, and urinary incontinence due to stress (SUI) [2]. It is a potent norepinephrine and serotonin reuptake inhibitor. No significant affinity exists between DLX and the cholinergic, dopaminergic, adrenergic, histaminergic, glutamate, opioid, or GABA receptors [3]. It helps to elevate mood, alleviate anxiety, promote sleep, and increase energy and appetite [4]. The drug is efficiently absorbed after oral administration, binds to protein 96%, reaches its peak plasma levels (C max) at a median time of 6 h, and has a mean elimination half-life of 12 h. DLX can be regarded as a selective reuptake inhibitor at the 5HT and NE transporters because it does not have any noticeable affinity for dopaminergic, cholinergic, histaminergic, opioid, glutamate, or GABA reuptake transporters. DLX is extensively metabolized, however the main circulating metabolites have little or no effect on the drug’s pharmacologic efficacy [5]. For the assay of DLX in pharmaceutical products, many approaches were employed, these methods include spectrophotometry [1, 2, 5, 6], spectrofluorimetry [3, 7–9], high-performance liquid chromatography (HPLC) [10–12], Thin-layer chromatography (TLC) [4, 13–15], potentiometry [16–18] and voltammetry [19–22].

Avanafil directly inhibits the cGMP-specific type 5 phosphodiesterase. It is employed for the treatment of erectile dysfunction [23]. Avanafil has the advantage of having a much faster beginning of effect than other PDE5 inhibitors. It is readily absorbed and reaches its peak concentration in 30–45 min [24]. The antiproliferative and vasodilatory effects of endogenous nitric oxide, which is excreted by endothelial cells that lining the arteries, are mediated by AVN by inhibiting phosphodiesterase type-5, which increases cGMP in the penile vasculature and helps guarantee a strong erection. AVN has been developed because it is more highly selective for the PDE5 isoenzyme than other inhibitors that target PDE5 [25]. Avanafil overdose symptoms include blurred vision, sudden hearing or visual loss, dyspnea, rapid pulsation. Its use is still preferred above that of the other PDE-5 medications, despite all of these adverse reactions. Avanafil has the following chemical name: (S)-4-[(3-chloro-4-methoxybenzyl)amino].-2-[2-(hydroxymethyl)-1-pyrrolidinyl]-N-(2-pyrimidinylmethyl)-5-pyrimidinecarboxamide (Fig. 1) [26]. AVN was given approval by the US FDA in 2012 and the European Medicines Agency did the same in 2013 [27]. Literature survey illustrates that a variety of techniques have been documented for the quantitative measurement of avanafil including spectrophotometry [28, 29], spectrofluorimetry [24, 26, 30], HPLC [23, 25, 31], LC/MS [32–34], TLC [35], voltammetry [36–38] and capillary electrophoresis [39]. Additionally, a recent review article was published which summarized most of the analytical methods for determination of sex stimulants including avanafil [40].

Medications for depression particularly selective serotonin reuptake inhibitors (SSRIs) and serotonin norepinephrine reuptake inhibitors (SNRIs) can cause sexual dysfunction as a side effect [41]. The unpleasant sexual side effects including erectile dysfunction can be treated with AVN. For the remedying of male reproductive problems induced by duloxetine, a number of physicians suggested a private prescription regarding phosphodiesterase-5 (PDE_5_) inhibitors like Avanafil [42, 43]. Therefore, the focus of our investigation was the simultaneous determination of DLX and AVN. The current study presents a novel, specific, and environmentally friendly HPTLC approach for the separation and quantification of both drugs in bulk, laboratory-prepared combinations, and human plasma with good precision, sensitivity, and selectivity results in accordance with ICH recommendations. This study is novel because there is right now no published HPTLC protocol for quantitative separation of DLX and AVN together.

## Experimental

### Instrumentation

The chromatographic analysis was performed using a CAMAG (Muttenz, Switzerland) TLC apparatus. To customize the data, Scanner 3 was operated by Vision CATS version software TLC. High-pressure mercury vapor lamps were employed as the scanner’s radiation source. The slit’s dimensions were 5 × 0.2 mm, and the scanning speed was 20 mm/s. The samples were applied to the plate using a Hamilton syringe (100 µL; Bonaduz, Switzerland) with a Linomat 5 auto-sampler while being lightly streamed with nitrogen. The development of the plate was carried out in a twin-trough chamber (27.0 × 26.5 × 7.0 cm) in the ascending mode.

### Chemicals are used and materials

DLX powder had been generously provided by Mash Premiere pharmaceutical company (Badr City, Cairo, Egypt), and AVN powder received via Andalous Pharma (6^th^ of October city, Cairo, Egypt). HPLC grade solvents (acetone and methanol) were purchased from Merck (Darmstadt, Germany) and 33% ammonia solution was obtained from El Nasr Pharmaceutical Chemical Co. (Cairo, Egypt).

### Preparation of standard solution

25 mg were weighed precisely of each medication, was dissolved in 10 mL of methanol via a sonicator before being diluted to a final volume of 25 mL with methanol to produce the stock solutions (1.0 mg/mL) for DLX and AVN separately. Following that, standard working solutions were produced by diluting the stock solutions using the same diluent to produce concentrations of 1, 10, 20, 40, 80, and 160 µg/mL for DLX, and 2, 4, 20, 40, 80, and 160 µg/ mL for AVN. Lastly, to acquire final concentrations, a small portion of Lastly, a small portion of 5 µl of each working solution was spotted onto the plate to reach final concentrations of DLX of 5, 50, 100, 200, 400, and 800 ng/spot and AVN of 10, 20, 100, 200, 400, and 800 ng/spot.

### Procedures

#### Chromatographic conditions

On HPTLC plates (Merck, Darmstadt, Germany) pre-coated with silica gel 60 _F254,_ all chromatographic investigations were performed. The plates were divided into 20 × 5-centimeter segments. The mobile phase was allowed to developed until reaching a migration distance of 3.5 cm from the origin [44–46]. For the purpose of preventing the drug spot from dissolving when the TLC plate was inserted inside the chamber of development, the band was positioned 1 cm farther away the lowest edge of the plate, with a width of 4 mm and a distance of 6 mm between each two subsequent bands displacing a 10 mm distance from the plate’s beginning. The bands were steamed with nitrogen to dry. Nitrogen drying prevents the band from widening, what benefits HPTLC by enabling the simultaneous assessment of numerous samples in a single run. Before starting chromatographic development, the plates that were used had previously been methanol-washed.

Methanol: acetone: 33% ammonia (8:2:0.05, v/v/v) utilized to be the mobile phase for chromatographic development. the chamber had been completely saturated with the mobile phase solvents for 20 min at the ambient temperature., and the mobile phase volume was actually 10 mL. Hair dryer was used to dry the plate and then it was canned at slit dimension of 5 × 0.2 mm with CAMAG TLC scanner at dual wavelength of 232 and 253 nm for DLX and AVN, respectively, in the absorbance reflectance mode.

#### Procedure for pharmaceutical preparations

Ten tablets of Erovanafile ® 200 mg were finely crushed. From this, a carefully weighed amount of tablet powder equivalent to 50 mg of AVN was transferred into a 50 mL volumetric flask. The contents of 10 capsules of Cymbatex® 30 mg were carefully mixed, and an exactly weighed quantity of powder equal to 50 mg DLX was added to the same flask. Subsequently, the drugs were extracted using methanol through sonication for 30 min. Then, the volume was adjusted to 50 ml using the same solvent after which the initial part of the filtrate was cast-off. The standard assay procedure was followed, involving five measurements for each concentration.

#### Procedure for spiked human plasma

Samples of blood from healthy participants were collected at Misr Hospital (Sohag, Egypt) via the forearm vein and then immediately put into heparinized tubes. The work’s purpose was explained to the volunteer before he signed his assent. The method for using human plasma samples was accepted by the Committee on Ethics of Scientific Research, Faculty of Pharmacy, Sohag University, and complied with the Declaration of Helsinki Recommendations [47]. In all cases, informed written consent was obtained from each participant before donating the blood samples. Centrifugation was used to separate the plasma for 30 min. at 4000 rpm. 1.0 ml of drug-free plasma, 1.0 ml of drug mixture solution with concentrations of 10, 40, and 80 µg mL^− 1^ for both drugs, and 2.0 ml of acetonitrile as a protein precipitator were added to a centrifugation tube. The sample was then thoroughly mixed. The tube was centrifuged at 4000 rpm for 30 min. The collected supernatant was clear and was analyzed in accordance with the steps outlined under “Chromatographic conditions.”

## Results and discussion

TLC is one of the most familiar and adaptable techniques used in detection and simultaneous quantification of several pharmaceuticals. It has several advantages including; simplicity, cost-effectiveness, rapidness as well as batch analysis with accurate quantification of multicomponent mixture [48, 49]. TLC method is an effective approach for simultaneously determining mixtures because it has great selectivity and accuracy, requires little sample preparation, and uses little solvent.

**Fig. 2:**
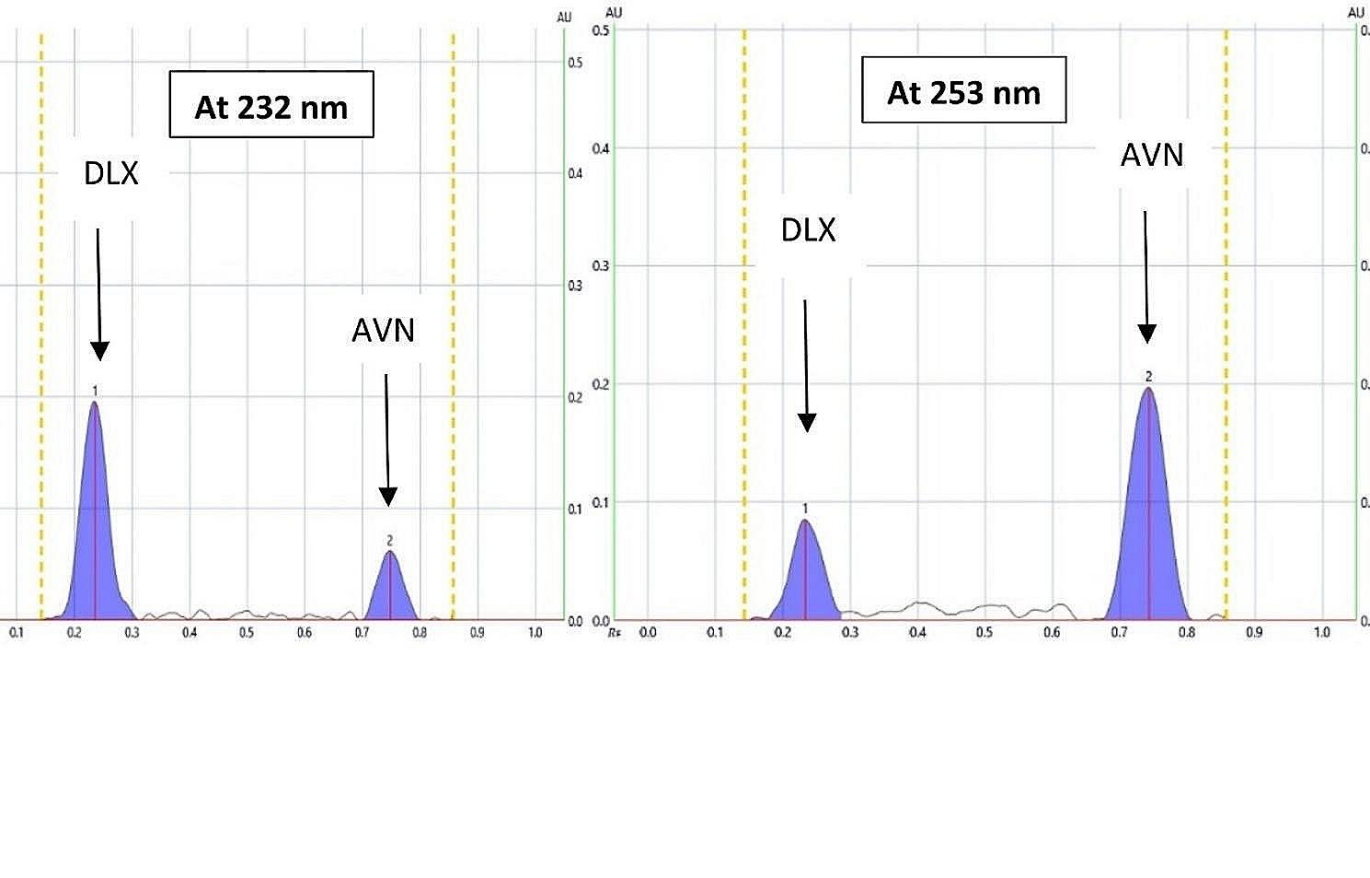
2D HPTLC densitogram of the mixture containing (400 ng/band) of DLX and (400 ng/band) of AVN measured at dual wavelength (232 nm for DLX and 253 nm for AVN)

Successful treatment of sexual dysfunction caused by DLX could be attained through simultaneous administration of AVN. Consequently, there is a critical need to establish an innovative method for the simultaneous determination of the examined medications. In the present TLC approach, a dual wavelength procedure was used that is highly sensitive, quick, simple, selective, and affordable. The absorption spectra for both medications are illustrated in (Fig. 2). It was possible to quantify several samples containing both DLX and AVN in a single run, which preserves time, chemical substances, and manpower. The suggested approach was able to measure both medications in human plasma that has been spiked without any interference from the plasma components. Thus, the method can be applied for the measurement of blood levels of the investigated medications following co-administration.

### Method development and optimization

Numerous variables were examined to determine their effects on the approach so as to achieve the best resolution and separation with appropriate values of retardation factor (Rf) and sharp symmetrical peaks. The mobile phase’s composition, saturation period of time, and scanning wavelengths have been adjusted as parameters affecting the suggested TLC-densitometric methodology.

#### Mobile phase composition

In order to separate the binary mixture of DLX and AVN, various mobile phase compositions investigated. Numerous trials for optimizing the mobile phase composition had been performed by adjusting the ratios of several organic solvents as shown in (Table 1). For example, methanol-acetone (8:2, v/v) was tried where DLX was very near to the base line with tailing. Toluene-methanol (7:3, v/v) could achieve good separation but with tailing. Upon using n-hexane-methanol (8:2, v/v), both DLX and AVN remained at the baseline. Additionally, when, acetone-ethanol-ammonia (8:1:1, v/v/v) were used, the two peaks were near the solvent front line. In addition, ethyl acetate-acetone-ammonia (8:1:1, v/v/v) could not well separate the two peaks. Finally, the optimum mobile phase which could efficiently separate DLX and AVN combination was methanol-acetone-ammonia (8:2:0.05, v/v/v) as shown in Fig. 2. The Rf values of the separated peaks were 0.23 for DLX and 0.75 for AVN, it was necessary to use 33% ammonia solution for the purpose of preventing bands from tailing. The AVN band appears close to the solvent front with higher ammonia volumes. Therefore, a mixture of methanol-acetone-33% ammonia solution (8:2:0.05, v/v/v) was used as the mobile phase that could attain the most effective separation between the two components.

**Table 1:** Different composition of mobile phases and the corresponding RF for the separation of DLX ‒ AVN binary mixture

Mobile phase	DLX *R*_F_	AVN *R*_F_
Methanol-acetone (2:8, v/v)	0.1 with tailing	0.7 with tailing
Methanol-acetone (8:2, v/v)	0.15 with tailing	0.7
N-hexane-ethyl acetate (2:8, v/v)	At baseline	0.1 with tailing
N-hexane-ethyl acetate (8:2, v/v)	At baseline	At baseline
Toluene-methanol (7:3, v/v)	0.3 with tailing	0.6
Ethyl acetate-acetonitrile (8:2, v/v)	At baseline	At baseline
Ethyl acetate l-acetone-ammonia (8:1:1, v/v/v)	0.33	0.27
Acetone-methanol-ammonia (8:1:1, v/v/v)	0.8	0.9
Acetone-ethanol-ammonia (8:1:1, v/v/v)	0.85	At solvent front
Ethyl acetate-toluene-methanol (7:2:1, v/v/v)	At baseline	0.17
Toluene-ethyl acetate-acetic acid (6:2:2, v/v/v)	0.2	At baseline
Methanol-acetone-ammonia (8:2:0.05, v/v/v)	0.23	0.75

#### Time of saturation

It is crucial to fully saturate the chamber where development is happening by adding the mobile phase. because it significantly affects chromatographic separation. Prior to chromatographic development, the development jar was permitted to get saturated with the mobile phase for varying periods of time (10–30 min). It was observed that 20 min was adequate to obtain good separation.

#### Scanning wavelength

**Fig. 3:**
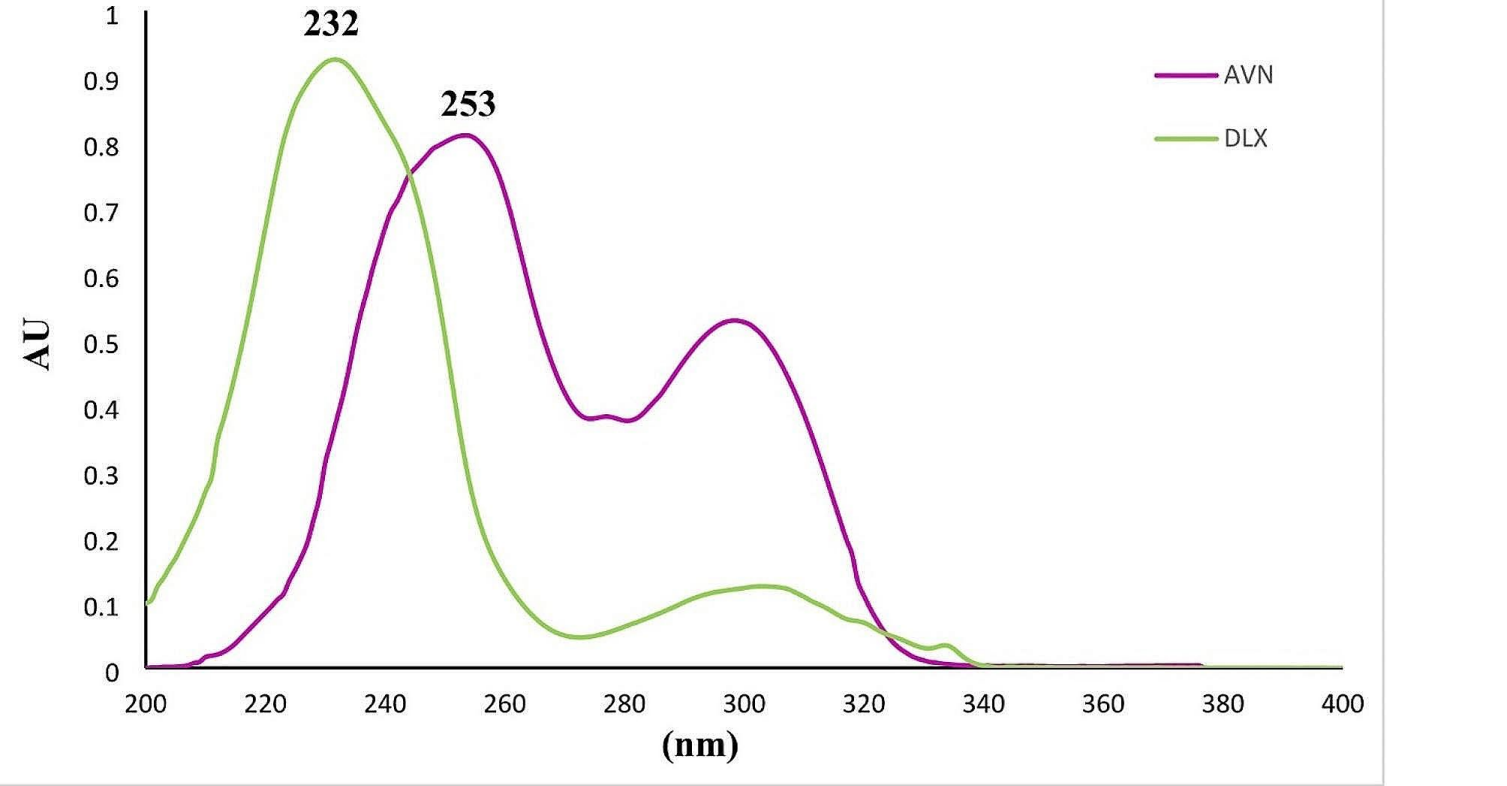
Absorption spectra of DLX and AVN (6 µg/mL) in methanol solvent

In order to determine the best wavelength for detecting both medications, a plate that had been spotted with both DLX and AVN was developed and then scanned in the UV region between 200 and 400 nm. The UV spectra of the suggested substances are shown in Fig. 3. The optimal scanning wavelengths that exhibit the best sensitivity and selectivity were 232 nm for DLX and 253 nm for AVN. The peaks that were produced are shown in (Fig. 2) and were sharp, symmetrical, well-separated, and noise-free.

### Method validation

The suggested TLC methodology has been validated to be compliant with the ICH guidelines [50]. The evaluated parameters were; the linearity range, detection and quantitation limits, accuracy, precision, and robustness. All results are presented as percentages.

#### Linearity

**Fig. 4:**
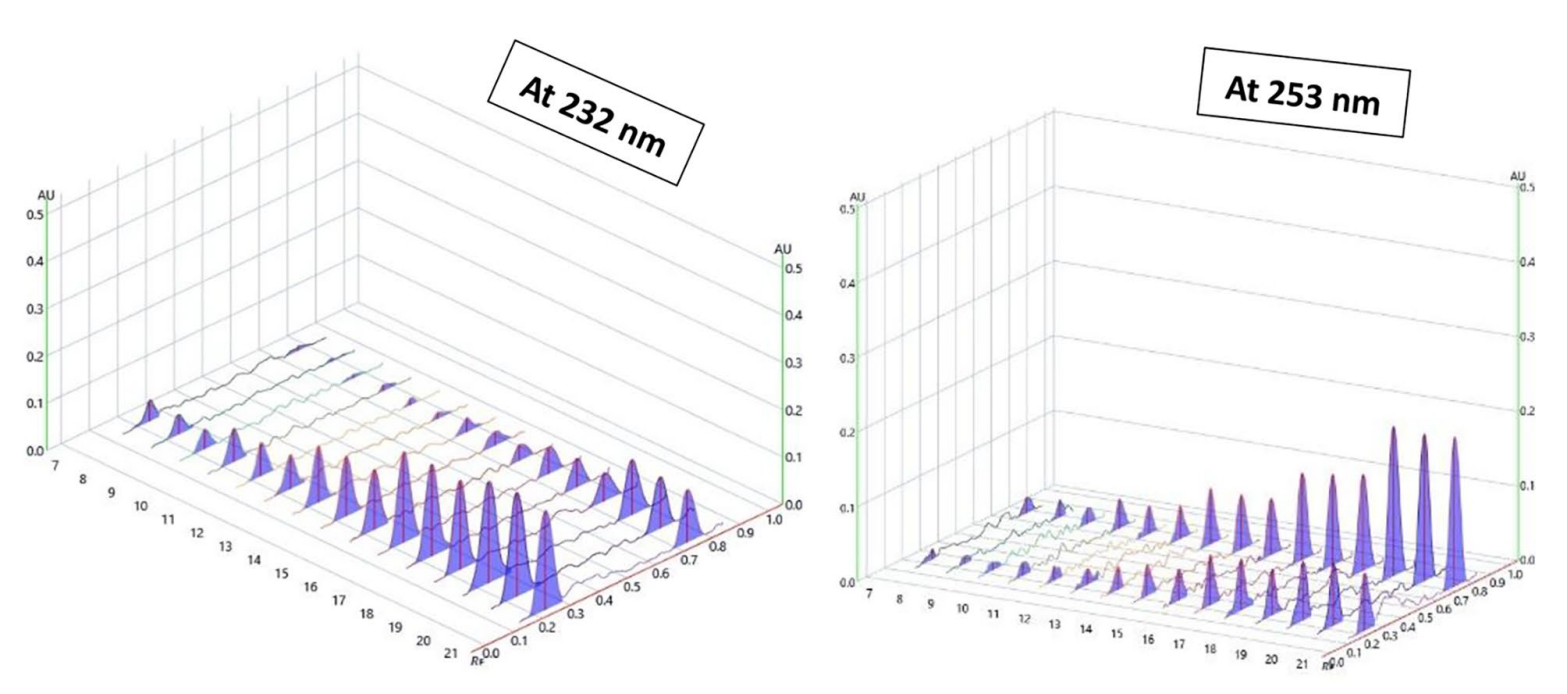
Three-dimensional HPTLC densitogram showing recorded intensities against Rf values (5–800 ng/band) for DLX and (10–800 ng/band) for AVN, measured at dual wavelength (232 nm for DLX and 253 nm for AVN)

Several medication solutions at varying concentrations were examined using the analysis procedure described above. The peak area values were recorded and displayed vs. drug concentrations in ng/spot for constructing the calibration curves after plate development and scanning at 232 and 253 nm. Three measurements were made for each concentration. It readily became apparent that a residual graph of a second order polynomial fit the data considerably much better than the linear regression model with higher values for the determination and correlation coefficient. The following polynomial equation was employed to fit the data; y = ax^2^ + bx + c. This relationship was proportionate within concentration ranges of 5-800 ng/spot (1–160 µg/mL) for DLX and 10–800 ng/spot (2–160 µg/mL) for AVN (Fig. 4). For the quadratic polynomial model fit, the estimated correlation and determination coefficients were found to be *r* = 0.9999 and r^2^ = 0.9999. It showed that the examined concentrations and the determined peak areas had a strong correlation. Table 2 displays the additional statistical variables for the second order polynomial regression equation and other validation parameters for both medications.

**Table 2:** Statistical data of some analytical parameters of the proposed method

Parameters	Avanafil	Duloxetine
Linear range (ng/spot)	10–800 (2–160 µg/mL)	5-800 (1–160 µg/mL)
Coefficient x ± SD	1.99E-05 ± 1.53E-07	2.33E-05 ± 8.68E-08
Coefficient x2 ± SD	-1.129E-08 ± 1.843E-10	-9.945E-09 ± 1.036E-10
Intercept ± SD	0.00043 ± 1.9E-05	0.0012 ± 1.09E-05
Correlation coefficient (r)	0.9999	0.9999
Determination coefficient (r^2^)	0.9999	0.9999
Number of determinations	5	5
Limit of quantitation (ng/spot)	9.53 (1.91 µg/mL)	4.69 (0.94 µg/mL)
Limit of detection (ng/spot)	3.15 (0.63 µg/mL)	1.55 (0.31 µg/mL)

#### Limits of detection and quantification

The limits of quantitation (LOQ) and detection (LOD) have been estimated to assess the sensitivity of the approach. The recommended approach was discovered to be sensitive due to the low values of LOD and LOQ for DLX and AVN, as shown in (Table 2). The coefficient of the X variable from the polynomial regression equation and the standard deviation of the intercept were used to determine LOD and LOQ. The formulas used were LOD = 3.3σ/S and LOQ = 10σ/S, where (S) is the coefficient of the x variable and (σ) is the standard deviation of the intercept. The calculated LOD values were 1.55 and 3.15 ng/spot (0.31 and 0.63 µg/mL) for DLX and AVN, respectively, and the calculated LOQ values were 4.69 and 9.53 ng/spot (0.94 and 1.91 µg/mL) for DLX and AVN, respectively (Table 2).

#### Accuracy

The recovery percentage of each of the drugs under study was calculated at 3 different concentrations, covering the linear range (low, medium, and high), after being determined by the recommended procedure. Three replicate assessments for each concentration were carried out in order to judge the accuracy of the suggested method. The obtained findings demonstrated the excellent accuracy of the suggested procedure for the reason that the standard deviation values were low and the estimated recovery value percentages were close to 100% (Table 3).

**Table 3:** Evaluation of accuracy of the analytical HPTLC procedure for determination of the investigated drugs

Drug	Conc. (ng/spot)		Amount found (ng/spot)	% Recovery ^a^ ± SD	
Avanafil	200		196.06	98.03 ± 2.07	
	400		401.88	100.47 ± 2.98	
	600		594.9	99.15 ± 0.18	
Duloxetine	200		201.62	100.81 ± 1.40	
	400		405.28	101.32 ± 1.24	
	600		608.16	101.36 ± 1.52	

^**a**^ Mean of three determinations, SD, standard deviation

#### Precision

Two types of precision, intra-day (repeatability) and inter-day (intermediate), were assessed for the proposed approach. The recommended approach was used to analyze a total of three concentrations covering the range of linearity (200, 400, and 600 ng/spot for both drugs) Three separate times on one day to evaluate the repeatability, while the suggested method was used to analyze three concentrations over three consecutive days to estimate the intermediate precision. As shown in Table 4, relative standard deviation (%RSD) values were fairly small.

**Table 4:** Evaluation of intra-day and inter-day precisions for the determination of the investigated drugs with the proposed method

Drug	Conc. (ng/spot)		intra-day precision % recovery ± SD ^a^		inter-day precision % recovery ± SD ^a^		
**Avanafil**	200		101.76 ± 2.22		101.69 ± 2.67		
	400		100.52 ± 1.43		99.26 ± 1.68		
	600		101.22 ± 0.89		100.64 ± 1.34		
**Duloxetine**	200		100.54 ± 1.69		101.81 ± 2.05		
	400		99.65 ± 1.17		98.59 ± 1.44		
	600		99.48 ± 1.94		100.47 ± 1.79		

^**a**^ Mean of three determination, SD, standard deviation

#### Robustness

Minor but intentional variations in the chromatographic procedure variables were made, and their influence on resulting data were examined, in order to evaluate the robustness of the proposed approach. Minor changes were made to the detection wavelength, saturation period of time, and constitution of the mobile phase system. According to the findings, none of the analyses of the two medications were significantly impacted by the modification made to the parameters under investigation. (Table 5). This demonstrated that the suggested approach was robust.

**Table 5:** Evaluation of the robustness of the proposed HPTLC method

Normal condition	Changed condition		AVN 400 (ng/spot)		DLX 400 (ng/spot)
			% recovery ± SD ^a^		% recovery ± SD ^a^
Mobile phase composition ethyl acetate-acetonitrile-ammonia (8:2:0.05, v/v/v)	(8.2:1.8:0.05, v/v/v)		98.79 ± 1.13		99.71 ± 1.94
	(7.8:2.2:0.05, v/v/v)		100.36 ± 2.07		100.57 ± 1.37
Wavelength					
AVN 253 nm	251 nm		98.26 ± 1.56		
	255 nm		101.7 ± 2.11		
DLX 232 nm	230 nm				99.74 ± 1.32
	234 nm				100.81 ± 2.12
Chamber saturation time 20 min	15 min		98.86 ± 1.67		99.65 ± 1.86
	25 min		100.45 ± 1.98		101.58 ± 1.55

^**a**^ Mean of three determination, SD, standard deviation

#### Specificity

As seen in the HPTLC chromatogram (Fig. 3) that shows entirely separation of DLX and AVN serves as a demonstration of the proposed method’s specificity. Additionally, the lack of anypeaks at the R_f_ of the investigated medication and the successful application of the technique to the synthetic mixture, as shown in (Table 6), demonstrate the absence of excipient impact.

**Table 6:** Estimation of DLX and AVN in their laboratory-prepared mixtures by the proposed HPTLC method

Parameter	Proposed method		Reported method	
	Duloxetine	Avanafil	Duloxetine	Avanafil
Number of measurements	5	5	5	5
Mean % recovery ^a^	101.24	99.63	101.62	98.27
%RSD	0.63	0.92	0.75	1.16
t-test ^b^	0.86	2.06		
F-value ^b^	1.42	1.59		

^a^ Mean of five measurements, %RSD, relative standard deviation

^b^ Tabulated value at 95% confidence limit, F = 6.388 and t = 2.306

### Application of the suggested approach

#### Application to pharmaceutical preparations

The suggested HPTLC approach was successfully applied to analyze laboratory-made solutions of the Erovanafile® tablets and Cymbalta (the commercial dosage forms in the local market). As indicated in (Table 6), the final results were compared to those attained using the previously published approaches [2] for DLX and [51] for AVN, as shown in Table 6. According to t and F tests, it was found that there was no statistically significant difference between the findings produced by the established method and those obtained by the methods that were published for the mixture ingredients. The findings obtained showed that there is no interference from either the co-administered medication or those frequently experienced additives and that the proposed approach is beneficial for the examined medications to achieve satisfactory recovery. The suggested approach is sensitive, specific, precise, and accurate. It is appropriate for determining the dosage forms of the investigated medications and for use in quality-control tests in laboratories.

#### Application to spiked human plasma

**Fig. 5:**
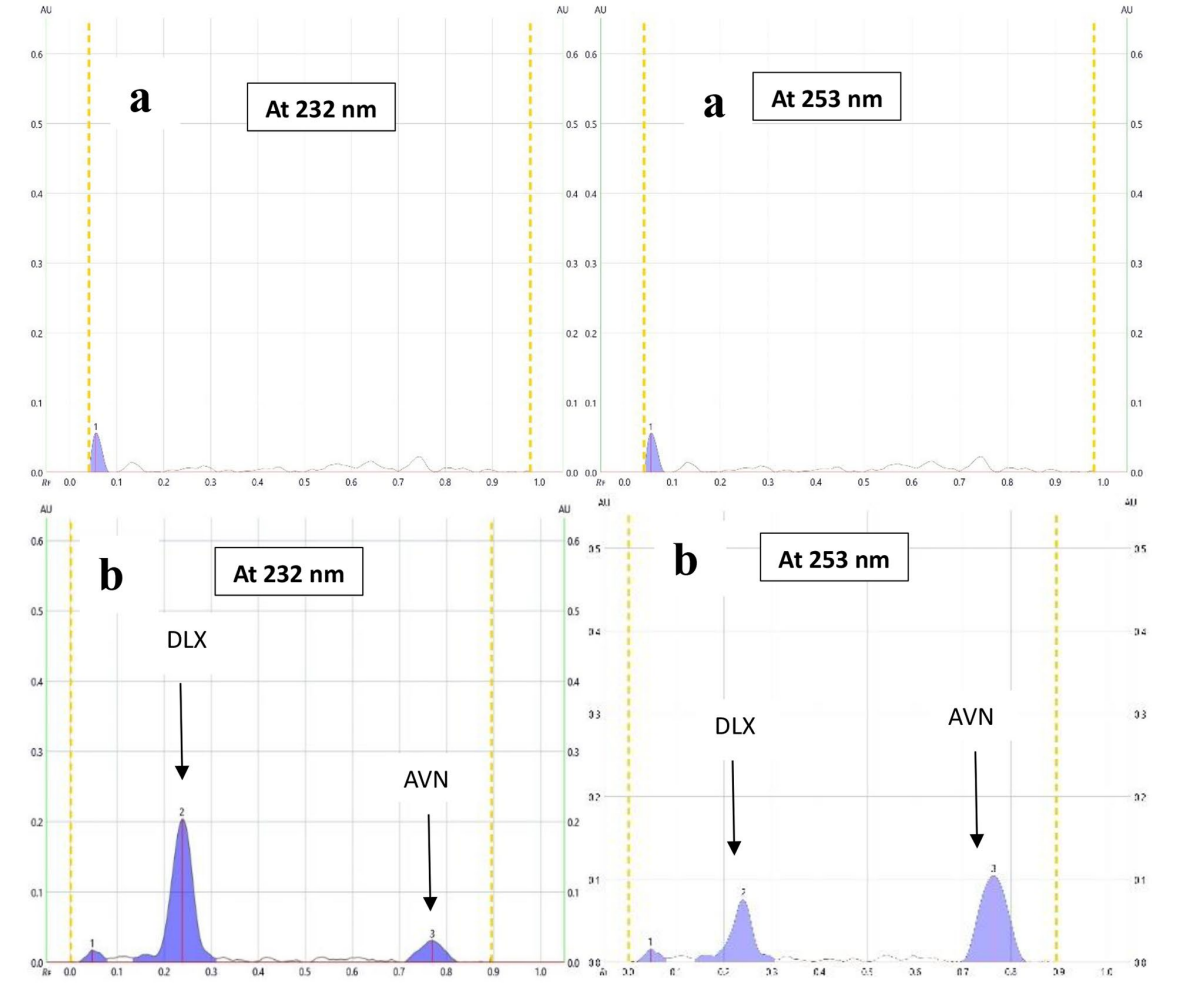
2D HPTLC densitogram of plasma sample (**a**) before and (**b**) after spiking with (400 ng/band) of DLX and (200 ng/band) of AVN measured at dual wavelength (232 nm for DLX and 253 nm for AVN)

The extremely sensitive HPTLC approach used in the current work allowed for an analysis of the DLX-AVN mixture in human plasma. The samples of plasma have been spiked with three different concentrations of the drug combination. The drugs concentrations were determined in accordance with the established protocol after a simple precipitation of proteins using acetonitrile and centrifugation. The chromatogram of the analyzed samples under investigation effectively provided acceptable identification and acceptable separation of the two drugs on the TLC plate, as demonstrated in (Fig. 5b). A sample of plasma that was not previously spiked with any drugs was also subjected to the procedure as a blank analysis, as seen in (Fig. 5a). The peaks of the plasma components are weak, well-resolved and completely separated from the peaks of the drugs. Consequently, the plasma components did not significantly interfere with the findings, which are shown in (Table 7). Because the found % recoveries were high enough it was suggested that the present technique is suitable for determining the considered drugs in plasma from humans after concurrent administration. Because collecting samples of blood from individuals receiving experimental medications was problematic, we preferred to conduct the research study using human plasma samples that had been prepared in vitro in combination with the medications under investigation. The results presented here showed that there is a minor variability that resulted from the influence of matrix n the estimation of DLX and AVN in human plasma.

**Table 7:** The application of the developed method to measure the drug in spiked human plasma

Drug	Conc. (ng/spot)		Amount found (ng/spot)		% Recovery ^a^ ± SD		
Avanafil	50		48.48		96.96 ± 1.88		
	200		196.1		98.05 ± 1.73		
	400		385.88		96.47 ± 1.18		
Duloxetine	50		47.72		95.43 ± 2.12		
	200		193.12		94.56 ± 2.38		
	400		391.68		97.92 ± 1.87		

^**a**^ Mean of three determination, SD, standard deviation

It should be noted that almost all the previously published methods were utilized to determined avanafil or duloxetine in their single form or in combination with other drugs. Thus, the merit of the present works comes from its ability to simultaneously determine both avanafil and duloxetine in presence of each other. However, recently a new spectrofluorimetric method was published for the simultaneous determination. Although the published method was more sensitive, the proposed method consumes less chemical and faster owing to its ability to analyze several samples in the same run. A summary comparing the proposed method with the reported methods is presented in the supplementary data (S1 and S2).

### Evaluation of method greenness

The three aspects that make up a “green” analysis are the absence of waste, the limited or absence use of hazardous chemicals, and the reduction in energy use. There are many tools available to assess how ecologically friendly the suggested approach is [52, 53]. The greenness profile, Eco-Scale methodology, and the Green Analytical Procedure Index (GAPI) were investigated to assess the greenness of the proposed TLC densitometric approach.

**Fig. 6:**
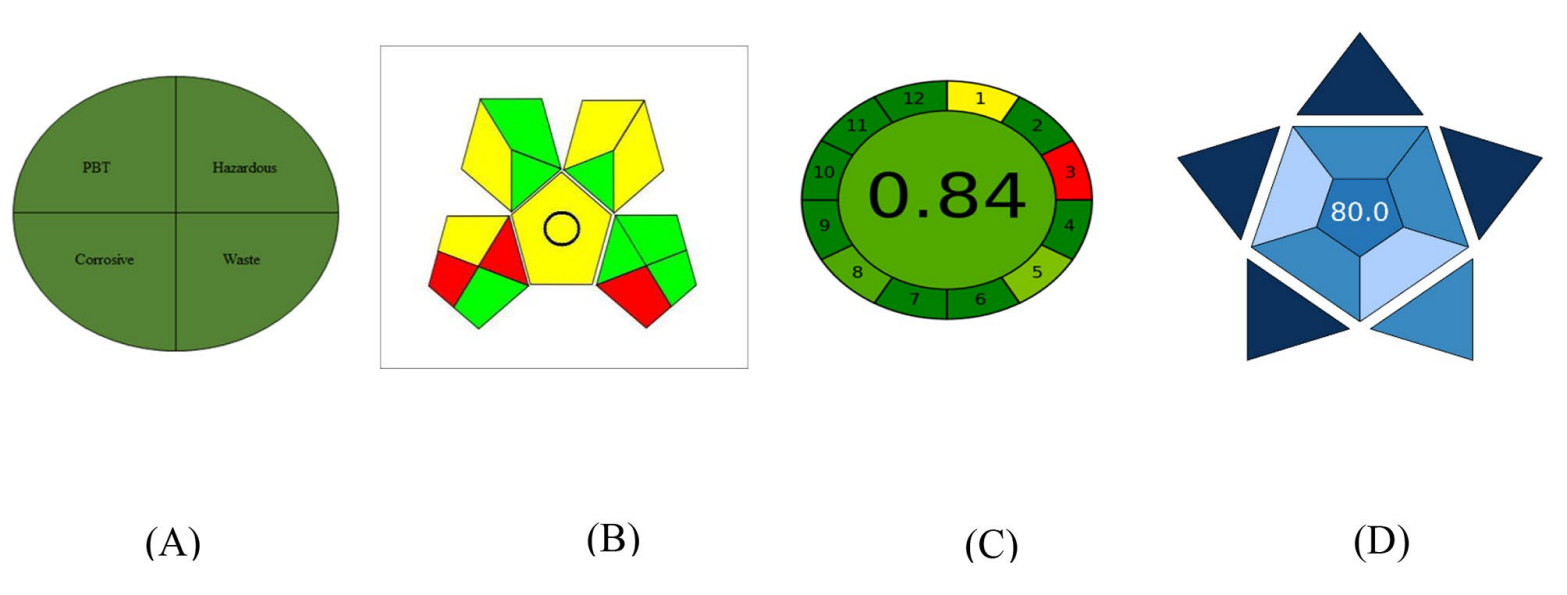
Evaluation of the proposed HPTLC method using NEMI pictograms (**A**), GAPI (**B**), AGREE (**C**), and BAGI (**D**) tools for greenness and blueness

The National Environmental Method Index (NEMI) was used to assess the established method’s greenness profile [54]. It depends on the use of PBTs, which are non-permanent, bio accumulative, and toxic solvents. The proposed methodology used acetonitrile and ethyl acetate as solvents, none of which are PBT. The pH of the developing system was also in the middle range of the pH scale therefore it was determined that it was not corrosive. The substances are safe to use, so not hazardous. Additionally, the waste volume was under 50 ml. Therefore, the procedure meets all the criteria for being a green procedure. Accordingly, the suggested TLC-densitometric methodology was deemed an environmentally green method as it passed all four greenness profile quadrants (Fig. 6).

Eco-scale is a simple method that is easily applied in the work of quality control laboratories. The analytical Eco-Scale score is calculated by utilizing the following equation (analytical Eco-Scale score = 100 − total penalty). Penalty points were assigned for each variable of the studied technique, including the amount of chemicals, workplace risks, waste, and energy consumption [55]. When the final score is greater than 75, the analytical strategy is regarded as an excellent green analysis. The solvents used in the current research were non-toxic. There was extremely little waste and very little energy utilized in the present study. These features allow the developed TLC-densitometric technique achieve an eco-Scale score of 86, which is an excellent level of environmental friendliness (Table 8).

**Table 8:** Evaluation of the greenness of the proposed HPTLC method using Eco scale score method

Parameters		Penalty points
Reagents	Methanol Acetone	6 4
Instrument		1
Energy consumption	(Less than 0.1 kWh per sample)	0
Occupational hazard	(Analytical process hermitization)	0
waste	(10 ml)	3
Total penalty points		14
analytical eco-scale total score		86

^a^ If the score is greater than 75, it represents excellent green analysis. If the score is greater than 50, it represents acceptable green analysis. If the score is less than 50, it represents inadequate green analysis

The Green Analytical Procedure Index (GAPI) is an additional tool that could be used to assess how environmentally friendly the suggested approach is [56]. Further characteristics of the analytical method are taken into account while evaluating the method’s greenness in this tool. To evaluate each step of the procedure of analysis that might have an effect on the environment, five pentagrams were generated. The three unique color codes of green, yellow, and red were used to represent minimum, medium, and major environmental implications, respectively. The GAPI pentagrams showed that the currently employed technique has an acceptable degree of greenness because it includes 7 green, 5 yellow, and 3 red shaded fields, as shown in Fig. 6.

For a deeper understanding of the unique green features of the suggested approach, a further green metric was included. This measure is referred to as the AGREE analytical tool [57]. The twelve sections that make up the AGREE framework are arranged in accordance with the twelve major GAC principles. Indeed, every component in these sections is given a distinct color that corresponds to its degree of greening, ranging from 0.0 (shown by the color red) to 1.0 (shown by the color green). This score is represented graphically as a clock-shaped pictogram in the middle of the framework, which functions as a color and score indicator for the whole procedure. The evaluation can be carried out with software, which automatically produced a report and a graph. The present methodology received an ultimate score of (0.84) upon evaluating all of these principles.

### Blueness evaluation

A new metric tool called the Blue Applicability Grade Index (BAGI) is utilized to assess the analytical method’s practicality [58]. Two different sets of results are produced by the BAGI metric tool: a graphical representation in the form of an asteroid pictogram and a numerical score at the center. The asteroid-shaped pictogram, which is made up of several hues of blue to represent varying degrees of compliance (dark blue for high, blue for moderate, light blue for low, and white for non-compliance), serves as a visual representation of the result of the evaluation. In order to develop a pictogram and a score that illustrate the practicality and functionality of an analytical approach, BAGI takes into account 10 factors (S3, Table). It is advised that the final score be greater than 60, so that the analytical method can be considered “practical”. As seen in the pictogram’s center, the suggested approach receives a high score of 80.0.

## Conclusion

The determination of DLX-AVN combination was performed for the first time using a precise, simple, ecologically friendly, and highly sensitive TLC approach. The evaluation of the proposed method was carried out using the Eco-scale, NEMI, GAPI, AGREE, and BAGI tools. The findings clearly demonstrate the sustainability and environmental friendliness of the presented approach. As a result, it can potentially be used in quality control laboratories. The technique uses a low-cost procedure with great precision and accuracy and is sensitive, straightforward, quick, selective, and doesn’t involve any complicated extraction steps. Without any noticeable interference from the plasma ingredients, the approach appeared sensitive enough to estimate the examined medications in plasma. For depressive patients, determining the concentrations of co-administered antidepressants and sexual stimulant medications in biological fluids is a crucial and important step. The recommended method offered a number of desirable benefits, including direct sample application for simplicity of use, quick analysis, a high number of samples per run, and minimal solvent use.

### Electronic supplementary material

Below is the link to the electronic supplementary material.

Supplementary Material 1

## Data Availability

The datasets used and/or analyzed during the current study are available from the corresponding author on reasonable request.
