# Supplementary material for: Simultaneous measurement of duloxetine hydrochloride and avanafil at dual-wavelength using novel ecologically friendly TLC-densitometric method: application to synthetic mixture and spiked human plasma with evaluation of greenness and blueness
Source: BMC Chem. 2024 May 3;18(1):92. doi: 10.1186/s13065-024-01195-2 (PMC11067093; doi:10.1186/s13065-024-01195-2)
Supplement: Supplementary file 1 — Supplementary Material 1 [file 13065_2024_1195_MOESM1_ESM.docx]

**S1: Comparison of the proposed method for Duloxetine determination with the Reported methods**

| **Technique used** | **Mobile phase** | **Linearity ng/ml** | **LOQ ng/ml** | **LOD ng/ml** | **Analyte(s)** | **Matrix** | **Ref.** |
| --- | --- | --- | --- | --- | --- | --- | --- |
| HPTLC-UV | methanol, acetone, and ammonia (8:2:0.05, V/V/V), | 5-800 | 4.69 | 1.55 | Duloxetine and Avanafil | Bulk and synthetic mixtures and spiked plasma | This work |
| HPTLC-UV | chloroform–ethyl acetate–benzene–33% ammonia solution (6:3:1:0.1, v/v) | 200–1400 | 71 | 23 | duloxetine hydrochloride and 1-naphthol | bulk and pharmaceutical formulation | [1] |
| HPTLC-UV | acetone-benzene-triethylamine (5:4.5:0.5, v/v) | 35–140 | 35 | 10 | Duloxetine | human serum. | [2] |
| HPTLC-UV | toluene-methanol-10% (*v/v*) ammonia 3:1.3:0.05(*v/v*) | 60–480 | 0.51 | 20 | duloxetine hydrochloride and olanzapine | a synthetic mixture | [4] |
| LC (C18) | aqueous 0.1% trifluoracetic acid, methanol, tetrahydrofuran (60: 20: 20, v/v/v) | 125000-375000 | 500 | 165 | Duloxetine | Impurities of duloxetine | [6] |
| UV visible spectrophotometric | | 10000-50000 | 1320 | 400 | Duloxetine | Pharmaceutical Formulations | [7] |
| UV visible spectrophotometric | | 5000-25000 | 2760 | 820 | Duloxetine | Capsule formulations | [8] |
| Spectrofluorometric method | | 100-1500 | 40 | 13 | Duloxetine and avanafil or tadalafil | Dosage form and human plasma | [9] |
| Spectrofluorometric method | | 100-2400 | 56 | 30 | Duloxetine | Capsule formulations | [10] |
| Spectrofluorometric method | | 1.0-70 | 1 | 0.5 | Duloxetine | Dosage forms | [11] |
| Electrochemical | | 1.0 × 10^−8^ to 1.0 × 10^−4^ mol L^−1^ | 1.0 × 10^−8^ mol L^−1^ | 3.0 × 10^−9^ mol L−1 | Duloxetine | commercial formulation and spiked human serum. | [12] |

**S2: Comparison of the proposed method for Avanafil determination with the Reported methods**

| **Technique used** | **Mobile phase** | **Linear range ng/ml** | **LOQ** | **LOD** | **Analyte(s)** | **Matrix** | **Ref.** | |
| --- | --- | --- | --- | --- | --- | --- | --- | --- |
| HPTLC-UV | methanol, acetone, and ammonia (8:2:0.05, v/v/v), | 10-800 | 9.53 | 3.15 | Duloxetine and Avanafil | Bulk and synthetic mixtures and spiked plasma | **This work** | |
| HPTLC-UV | chloroform: methanol: ethyl acetate: glacial acetic acid (5:2:3:0.2, v/v/v/v) | 1040-3640 | 484 | 160 | avanafil and dapoxetine hydrochloride | tablet dosage form | [13] | |
| HPLC (C18) | 0.1MAmmonium acetate buffer pH 2.5, methanol and acetonitrile with ratios (20:40:40) | 10000- 1000000 | 10 | 2 | avanafil | pharmaceutical dosage forms | [14] | |
| HPLC (C18) | acetonitrile: 0.15% triethylamine (40:60, v/v) at pH = 4.0 | 50–40000 | 130.6 | 43 | avanafil and dapoxetine hydrochloride | bulk powder, tablets and spiked human plasma | [15] | |
| UV chemometrics | | 1000-9000 | 1932 | 638 | avanafil and dapoxetine hydrochloride | Binary mixture & pharmaceutical product | [16] |  |
| spectrofluorimetric technique | | 100-1500 | 66.7 | 22 | Duloxetine and avanafil or tadalafil | Dosage form and human plasma | [9] |  |
| spectrofluorimetric technique | | 500-16000 | 225 | 75 | avanafil and dapoxetine hydrochloride | Pharmaceutical product & human plasma | [17] |  |
| spectrofluorimetric technique | | 500-1800 | 40 | 10 | Avanafil | Pharmaceutical dosage form | [18] |  |
| LC-MS/MS | | 0.25–25.0 | 0.19 | 0.06 | sildenafil, tadalafil, vardenafil and avanafil | Human plasma & urine | [19] |  |
| Electrochemical | | 0.10–6.0 μmol L^−1^ | 0.10 μmol L^−1^ | 0.035 μmol L^−1^ | avanafil and doxorubicin | real rabbit plasma | [20] |  |
| Electrochemical | | 5000-80000 | 1585 | 523 | avanafil and dapoxetine hydrochloride | combined pharmaceutical preparation. | [1] |  |

**S3** **(Table) The 10 factors utilized in evaluation of the proposed method using Blue Applicability Grade Index (BAGI)**

| **Parameter** | **Rating** | **Remarks** |
| --- | --- | --- |
| 1. Type of Analysis | moderately blue | Method is categorized as quantitative. |
| 1. Multi-Analyte Procedure | light blue | It determines two components. |
| 1. Analytical Technique Used | moderate blue | Densitometric equipment is an instrument that is easily accessible in most labs |
| 1. Simultaneous Sample Preparation | light blue | The suggested method's simultaneous preparation's ease of use and time-saving nature. |
| 1. Sample Preparation | moderately blue | Sample preparation is simple and inexpensive |
| 1. Samples Per Hour | dark blue | High number of samples (Batch analysis) |
| 1. Availability of Reagents | dark blue | there are no derivative reagents—common reagents that are available commercially |
| 1. Preconcentration | dark blue | it doesn't require preconcentration. |
| 1. Automation of Device | dark blue | Semi-automated instrument |
| 1. Amount of Samples | moderate blue | Sample is less than 100 µL |
